# Subfertile Chinese patients with diminished ovarian reserve: An analysis of pregnancy outcomes of ART cycles

**DOI:** 10.12669/pjms.39.2.6226

**Published:** 2023

**Authors:** Zeng-Yan Wang, Sun-Xing Huang, Jing-Di Yang, Dan-Ping Li, Yan-Wen Xu

**Affiliations:** 1 Zeng-Yan Wang, MD., Center for Reproductive Medicine, First Affiliated Hospital of Sun Yat-sen University, The Key Laboratory for Reproductive Medicine of Guangdong Province, Guangzhou, Guangdong, 510080, China; 2 Sun-Xing Huang, MD., Center for Reproductive Medicine, First Affiliated Hospital of Sun Yat-sen University, The Key Laboratory for Reproductive Medicine of Guangdong Province, Guangzhou, Guangdong, 510080, China; 3 Jing-Di Yang, MD., Center for Reproductive Medicine, First Affiliated Hospital of Sun Yat-sen University, The Key Laboratory for Reproductive Medicine of Guangdong Province, Guangzhou, Guangdong, 510080, China; 4 Dan-Ping Li, MD., Center for Reproductive Medicine, First Affiliated Hospital of Sun Yat-sen University, The Key Laboratory for Reproductive Medicine of Guangdong Province, Guangzhou, Guangdong, 510080, China; 5 Yan-Wen Xu, MD., Center for Reproductive Medicine, First Affiliated Hospital of Sun Yat-sen University, The Key Laboratory for Reproductive Medicine of Guangdong Province, Guangzhou, Guangdong, 510080, China

**Keywords:** Reproductive Techniques, Diminished Ovarian Reserve, Insemination, Artificial, In vitro fertilization, Infertility, Pregnancy Outcome

## Abstract

**Objective:**

To analyze the pregnancy outcomes of patients presenting with infertility solely due to diminished ovarian reserve (DOR) and treated by assisted reproductive technology (ART), including artificial insemination by husband (AIH) and *in vitro* fertilization (IVF).

**Methods:**

This was a retrospective study of subfertile patients due to DOR attending the Center for Reproductive Medicine in Guangzhou, China, between January 2010 and October 2015. Patients were assigned into either the AIH or IVF group. Within each group, these patients were further subgrouped based on their serum basal follicle-stimulating hormone (bFSH) level (10 ≤ bFSH ≤ 12IU/L and bFSH > 12IU/L) and age (20–30, 31–35, 36–40, and 41–45 years). The live birth rates were compared among these groups and subgroups.

**Result:**

A total of 1,003 patients with a median age of 38.91 (21–45) years were enrolled in the study. The live birth rate following AIH was 5.61% (25/446), which was significantly lower than that following IVF (25.13%; 140/557). In the subgroup analysis, the cumulative live birth rates in AIH group were significantly lower than those in the IVF groups (in the 10–12 IU/L bFSH subgroup, 13.74% vs. 41.13% (P<0.05) for patients aged ≤35 years, and 4.82% vs. 19.77% (P<0.05) for patients aged >35 years; in the >12 IU/L bFSH subgroup, 9.52% vs. 29.91% (P<0.05) for patients aged ≤35 years, and 5.71% vs. 20.55% (P<0.05) for patients aged >35 years). Longitudinal analysis showed that majority of live births, in AIH or IVF groups, were achieved in the first two cycles.

**Conclusions:**

In subfertile women with DOR, live birth rates following AIH were significantly lower than IVF, especially for the aged women. Considering the low efficacy of AIH and that majority of live births were achieved in the first two cycles, we suggest no more than two AIH treatment attempts for the aged women with DOR.

## INTRODUCTION

Diminished ovarian reserve (DOR) refers to significant reductions in ovarian volume and follicle pool reserve,^1^ thus contributing to poor oocyte quality and infertility.^2^,^3^ Various etiologies can lead to DOR, with aging being the most common cause.^4^ There is currently no uniform definition for DOR. Many studies proposed a diagnosis of DOR with an antral follicle count (AFC) <5–7, and/or serum anti-Müllerian hormone (AMH) <0.5–1.1 ng/mL,^5^,^6^ or the basal FSH≥10IU/L.^7^,^8^ The prevalence of DOR was estimated at about 10–25% among women seeking infertility treatment.^9^,^10^

As patients with DOR are characterized with a reduced ovarian response to gonadotropin stimulation, they commonly have poor outcomes with assisted reproductive technology (ART) treatments, including artificial insemination by husband (AIH), and *in vitro* fertilization (IVF) with or without intracytoplasmic sperm injection (ICSI).^11^ Therefore, DOR remains to be a challenge in the field of reproductive medicine. Very few published studies specifically targeted the treatment options for women with infertility solely due to DOR. One randomized clinical trial by Goldman *et al*. compared artificial insemination by the husband (AIH) with IVF/ICSI (intracytoplasmic sperm injection) treatment in aged women with unexplained infertility.^12^ The authors concluded that IVF was superior based on the fewer treatment cycles required and the higher pregnancy rate. However, a recent study^13^ in patients with low AMH level found that IVF and AIH had comparable pregnancy outcomes, and neither achieved satisfactory results. Hence, further studies are required to improve pregnancy outcomes in patients with infertility solely due to DOR.

Maternal age plays a pivotal role in determining reproductive outcomes. In the study of treatment options for DOR patients, pregnant women with different ages should be analyzed separately for the cumulative live-birth rate. In the present study, we compared the efficacy of IVF with AIH among patients of different ages and serum basal follicle-stimulating hormone (bFSH) levels. Our study may provide valuable information for determination of treatment options for patients with infertility solely due to DOR.

Our objective was to analyze the pregnancy outcomes of patients presenting with infertility solely due to diminished ovarian reserve (DOR) and treated by assisted reproductive technology (ART), including AIH and IVF.

## METHODS

We performed a retrospective study on subfertile patients due to DOR from the Center for Reproductive Medicine in Guangzhou, China between January 2010 and October 2015. Hospital medical records were reviewed. Patients who had a bFSH level ≥10 IU/L and with at least one patent fallopian tube were included in the present analysis. Patients were excluded if the husband had severe male infertility factors, such as serious oligozoospermia, asthenozoospermia, or azoospermia. The study was approved by the institutional ethical board of the First Affiliated Hospital of Sun Yat-sen University in 2018 (approval number [2018]281). Informed consent was waived due to the retrospective design of the study.

### Study protocol and data collection:

Venous blood was collected from the patients on the 2^nd^ to 5^th^ day of their menstrual cycles. The serum bFSH level was detected using a chemiluminescence kit (Beckman Coulter, USA) in the hospital laboratory.

All patients were separated into two groups: one group with a bFSH level of 10–12 IU/L and the other group with a bFSH level >12 IU/L. As the ovarian reserve decreases naturally with age, the patients in each group were further separated into four subgroups by age: 20–30 years, 31–35 years, 36–40 years, and 41–45 years. Primary outcome in this study was the cumulative live birth rate. Secondary outcomes included live birth rate, multiple pregnancies, miscarriage rate, and ectopic pregnancy rate.

The live birth rate by the initiating treatment referred to the live birth rate of the patients, whose first treatments in our center were AIH or IVF/ICSI. Live birth rate of the fist IVF/ICSI cycle included the fresh cycle and the after-all FET (frozen-thawed embryo transfer) cycles of frozen embryos from the same IVF/ICSI cycle. AIH cycle cumulative live birth rate referred to the live birth rate of patients with the first AIH cycle and the after-all AIH cycles; IVF/ICSI cycle cumulative live birth rate referred to the cumulative live birth rate of patients with the first IVF/ICSI cycle and the after-all IVF/ICSI cycles and FET cycles.

### Statistical methods:

SPSS v21.0 software (SPSS Inc., Chicago, IL, USA) was used for all statistical analyses. Continuous data were compared by the *t*-test, depending on the normality Kolmogorov–Smirnov test result. A Chi-squared test was used to compare the live birth rate after the first fertility treatment and the cumulative live birth rate. A Student’s *t*-test was used to compare the required number of treatment cycles to achieve a live birth, as well as the rate of multiple pregnancies between IVF/ICSI and AIH treatments. A P-value <0.05 was considered statistically significant.

## RESULTS

A total of 1,003 patients with a median age of 38.91 (21–45) years were enrolled in the study. The median number of AFC was 3.03 (0–8). The mode of ART used for patients with DOR included AIH and IVF/ICSI. Among 446 patients treated with AIH, 29 became pregnant, with a clinical pregnancy rate of 6.5% and a live birth rate of 5.61% (25/446). Among 557 patients treated with IVF/ICSI, 184 became pregnant, with a clinical pregnancy rate of 33.03% and a live birth rate of 25.13%(140/557) (Table-I).

**Table-I:** Clinical outcomes of patients with diminished ovarian reserve stratified by bFSH levels and age.

bFSH (IU/L)	Age (years)	Treatments	Clinical pregnancy rate (%)	Miscarriage rate (%)	Ectopic rate (%)	Live birth rate (%)
10 ≤ bFSH ≤ 12 (736)	20–30	AIH	7.54 (9/115)	11.1 (1/9)	11.1 (1/9)	6.09 (7/115^1^)
		IVF/ICSI	39.77 (35/88)*	8.57 (3/35)	2.86 (1/35)	30.09 (30/88^2^)*
	31–35	AIH	10.0 (13/130)	30.77 (4/13)	15.38 (2/13)	6.92 (9/130)
		IVF/ICSI	43.92 (65/148)*	18.46 (12/65)	3.08 (2/65)	33.78 (50/148^3^)*
	36–40	AIH	4.05 (3/74)	0 (0/74)	0 (0/74)	4.05 (3/74)
		IVF/ICSI	26.09 (30/115)*	40 (12/30)	3.33 (1/30)	13.91(16/115^4^)*
	41–45	AIH	0 (0/26)	–	–	0 (0/26)
		IVF/ICSI	17.5 (7/40)*	14.29 (1/7)	0 (0/40)	15 (6/40)*
bFSH >12 (267)	20–30	AIH	0 (0/25)	–	–	0 (0/25)
		IVF/ICSI	43.33 (13/30)*	0 (0/13)	7.69 (1/13)	40 (12/30)*
	31–35	AIH	5.89 (3/51)	0 (0/3)	33.3 (1/3)	3.92 (2/51)
		IVF/ICSI	30.19(16/53)*	12.5 (2/16)	6.25 (1/16)	24.53 (13/53)*
	36–40	AIH	9.99 (1/11)	0 (0/11)	0 (0/11)	9.99 (1/11)
		IVF/ICSI	32.08 (17/53)*	17.65 (3/17)*	5.89 (1/17)	24.53 (13/53)*
	41–45	AIH	0 (0/14)	–	–	0 (0/14)
		IVF/ICSI	3.33 (1/30)	100 (1/1)	–	0 (0/30)

* Significant difference between the AIH and IVF/ICSI groups (P<0.05). The number of cases in each group less than 30 was presented as the actual number of cases rather than the rates.

1 one case lost during the follow-up period;

2 one case of twins lost during the follow-up period;

3 one case of twins lost during the follow-up period;

4 one case lost during the follow-up period. bFSH, basal follicle-stimulating hormone; AIH, artificial insemination by husband; ICSI, intracytoplasmic sperm injection; IVF, in vitro fertilization.

Clinical outcomes of patients stratified by bFSH levels and age are shown in Table-I. There were 736 patients with a bFSH level of 10–12 IU/L and 267 patients with a bFSH >12 IU/L. In every age group with patients having 10–12 IU/L bFSH, the clinical pregnancy rate and live birth rate in patients treated by AIH were significantly lower than those in the IVF/ICSI group. Similarly, in every age group (except 41–45 years) with patients having >12 IU/L bFSH, the clinical pregnancy rate and live birth rate treated by AIH were significantly lower than those in the IVF/ICSI group.

The miscarriage rate and ectopic pregnancy rate were similar between the AIH and IVF/ICSI groups in the above subgroups except for those patients in the bFSH >12 IU/L subgroup aged 36–40 years [the miscarriage rates were 0% (0/11) *vs*. 17.65% (3/17), *P*<0.05].

### Longitudinal analysis of patients undergoing multiple treatments:

We further performed a longitudinal analysis of the patient outcomes. The data were sub-grouped using a cutoff age of 35 years and bFSH level of 12 IU/L, to ensure sufficient numbers of patients in each group for the statistical analysis. The outcomes are shown in Table-II. Apart from the number of cycles to achieve a live birth, in which there was no difference between the AIH and IVF treatments, all other measurements showed statistically significant differences between the AIH and IVF treatments.

**Table-II:** Longitudinal analysis of the pregnancy outcomes in subgroups of patients with diminished ovarian reserve.

bFSH(IU/L)	Age (years)	Factors	AIH	IVF	OR (95%CI)	P
10–12	≤35	Live birth rate by the initiating treatment (%)	7.14 (13/182)	35.45 (106/299)	0.141 (0.073–0.275)	<0.05
		Cumulative live birth rate (%)	13.74 (25/182)	41.13 (123/299)	0.216 (0.127–0.369)	<0.05
		Number of cycles to achieve live birth. N (0–n) and Mean ± SD	32 (1–3) 1.6 ± 0.75	119 (1–3) 1.55 ± 0.414		>0.05
		Multiple pregnancy rate (%)	4 (1/25)	39.83 (49/123)		<0.05
	>35	Live birth rate by the initiating treatment (%)	3.61 (3/83)	15.7 (27/172)	0.181(0.041–0.802)	<0.05
		Cumulative live birth rate (%)	4.82 (4/83)	19.77 (34/172)	0.211(0.061–0.728)	<0.05
		Number of cycles to achieve live birth. N (0–n) and Mean ± SD	4 (1–2) 1.33 ± 0.57	31 (1–3) 1.24 ± 0.52		– >0.05
		Multiple pregnancy rate (%)	0	29.41 (10/34)		<0.05
bFSH >12	≤35	Live birth rate by the initiating treatment (%)	4.76 (2/42)	23.08 (27/117)	0.162 (0.037–0.712)	<0.05
		Cumulative live birth rate (%)	9.52 (4/42)	29.91 (35/117)	0.242 (0.081–0.728)	<0.05
		Number of cycles to achieve live birth. N (0–n) and Mean ± SD	7 (1–3) 1.7 ± 0.95	54 (1–2) 1.28 ± 0.51		– >0.05
		Multiple pregnancy rate (%)	0	20 (7/35)		<0.05
	>35	Live birth rate by the initiating treatment (%)	2.86 (1/35)	17.81 (13/73)	0.099 (0.013–0.762)	<0.05
		Cumulative live birth rate (%)	5.71(2/35)	20.55 (15/73)	0.157 (0.035–0.698)	<0.05
		Number of cycles to achieve live birth. N (0–n) and Mean ± SD	3 (1–2) 1.5 + 0.707	27 (1–3) 1.23 + 0.52		– >0.05
		Multiple pregnancy rate (%)	0	33.33 (5/15)		<0.05

bFSH, basal follicle-stimulating hormone; AIH, artificial insemination by husband; IVF, *in vitro* fertilization.

## DISCUSSION

Both AIH and IVF/ICSI are the treatment options to assist pregnancy in infertile patients solely due to DOR. However, the live birth rate by AIH was significantly lower than that by IVF/ICSI (5.61% vs 25.13%) in our study. Considering that majority of live births were achieved in the first two cycles in both AIH and IVF/ICSI, we suggest no more than two AIH treatment attempts for the aged women with DOR.

Subfertile women commonly suffer from physical and psychiatric stress, and the financial pressure to get pregnant.^14^-^17^ Although the treatment strategies for unexplained infertility typically involve AIH and IVF,^18^-^20^ unfortunately, less than one-tenth of couples could achieve a live birth following the AIH treatment. Most of these couples still required IVF to have live births.^21^ The advantage of AIH include no need for oocyte pick-up and embryo culture in vitro, which is more natural and safer with lower cost, but the overall pregnancy rate is low. In addition, AIH might have a negative effect on sexual function.^22^ IVF has a higher pregnancy rate, however, it is more complex and costly with long-term safety concerns about the offspring.

The choice of an efficient treatment strategy is important for these subfertile couples. To our knowledge, very few studies have compared AIH with IVF regarding subfertile couples solely due to DOR among different age groups. The results of a randomized controlled study (RCT) showed that, the cumulative pregnancy rate of young subfertile women with normal ovarian function who had three cycles of AIH could reach 31%, significantly higher than expected management (9%).^23^ In another RCT with 207 young subfertile couples randomly assigned to three cycles of AIH (n=101) or one cycle of IVF (n=106), the results suggested that the live birth rate for the AIH group was 24.7%, not significantly different from the IVF group (31.1%).^24^ The patients in these studies were young women with normal ovarian reserve who were commonly offered for AIH in the clinical practice.

Our data showed that even for the young DOR women with unexplained infertility, the live birth rates of AIH were very low, with 6.09% in the 20-30 years group and 6.92% in the 31-35 years group, which were significantly lower than that in the IVF/ICSI group (30.09% and 33.78%, respectively). The results were consistent with the previous study about the younger DOR patients.^25^

In order to analyze the efficiency of AIH or IVF for subfertile DOR women, we compared the cumulative live birth rate of patients who chose AIH or IVF in the initiating treatment cycle. Our results showed that the cumulative live birth rates of women aged ≤35 years by the initiating treatment in the AIH subgroups were significantly lower than those in the IVF subgroups (in the 10–12 IU/L bFSH group, 13.74% vs. 41.13%, P<0.05; in the >12 IU/L bFSH group, 9.52% vs. 29.91%, P<0.05). These results indicated that direct IVF was more efficient for young DOR women with unexplained infertility. However, our study was a retrospective analysis. Many patients switched to IVF after 1-2 cycles of AIH, which might lead to biased results. Therefore, further prospective studies are needed to determine which strategy is more effective young DOR women with unexplained infertility.

We also compared the IVF and AIH efficacy in women with advanced ages. Aged DOR women also have reduced egg quality and an increased likelihood of aneuploidy.^26^ A previous study had shown that aged DOR women had low pregnancy and live birth rates, as well as a high abortion rate.^27^ In a RCT study of aged women with unexplained infertility in 2014, 154 couples randomized to receive AIH (103) or direct IVF (51). The cumulative clinical pregnancy rates per couple after the first two cycles of AIH or directly IVF were 19.54% and 49.0%, respectively. Majority live-born infants (84.2%) were achieved *via* IVF.^12^ This study demonstrated that direct IVF could achieve superior pregnancy rate with fewer treatment cycles.^12^ Our results were similar to a previous study,^12^ with the cumulative live birth rates of women aged >35 years in the initial AIH cycle significantly lower than those in the IVF cycles, indicating that direct IVF should be considered for these women.

Because the higher bFSH level was associated with the lower ovarian reserve,^7^,^8^ we stratified the patients into two subgroups according to the bFSH level (10≤bFSH<12mIU/ml and bFSH≥12mIU/ml) to compare the effects of slight and severe decrease in ovarian reserve on pregnancy outcomes. Our results showed that, among young women (≤35 years old), the cumulative live birth rate of AIH in the slightly decreased ovarian reserve group(10≤bFSH<12mIU/ml) was significantly higher than that in the severely decreased ovarian reserve group (bFSH>12mIU/ml) (13.74% and 9.52%, respectively, P<0.05). The cumulative live rate in IVF was also significantly higher (41.13% and 19.77%, respectively, P<0.05). In the aged women (>35 years old), the cumulative live birth rate of AIH in the slightly decreased ovarian reserve group was similar to that in the severely decreased ovarian reserve group (4.82% and 5.71%, respectively, P>0.05). The cumulative live rate in IVF was also similar (19.77% and 20.55%, respectively, P>0.05). Our results showed that the live birth rates of AIH and IVF in young DOR women with unexplained infertility were significantly reduced with the increase of bFSH level. In aged women DOR women with unexplained infertility, the live birth rates of both AIH and IVF were very low regardless the ovarian reserve severity. For the aged women with DOR, the live birth rate would be affected even if their ovarian reserve were slightly reduced, and they need active treatment, including direct IVF.

To answer the question that how many cycles be performed to achieve a live birth, we performed longitudinal analysis of the patient outcomes. Our data also suggested that the number of treatment cycles that could achieve a live birth by AIH was similar to that achieved by IVF. This result might be influenced by physicians’ experiences in the treatment of DOR patients, and patients’ subjective choice of AIH or IVF. Some patients were likelier to request IVF/ICSI, which may lead to selection bias. Considering most live births were achieved in the first two cycles of both AIH and IVF/ICSI cycles and very low live birth rate in the aged women with AIH, we suggested that no more than two AIH attempts for this group of patients.

### Strength of the study:

A strength of our study is its large sample size, which allowed us to organize patients based on their different bFSH levels and ages.

### Limitations of the study:

It includes its retrospective design and single-center research. In addition, serum anti-Müllerian hormone (AMH) level, which is another marker for DOR, was not available in our patients. Hence, further prospective randomized studies are required to confirm our results.

## CONCLUSION

In conclusion, among patients with infertility solely due to DOR, live birth rates following AIH were significantly lower than IVF/ICSI, especially for the aged women. For aged subfertile DOR women, direct IVF was more effective than AIH, and no more than two AIH attempts should be performed. Based on our retrospective analysis results, for subfertile couples solely due to DOR, physicians can inform them about the estimated pregnancy rate and live birth rate of AIH and IVF in different age groups. Combining the patient’s own conditions and economic capacity, our results may be helpful for the physicians and patients to choose the appropriate treatments.

### Note:

The manuscript was presented at the 14th National Conference on Obstetrics and Gynecology of the Chinese Medical Association.

### Availability of data and materials:

The datasets generated and analyzed during the current study are available from the corresponding author on reasonable request.

### Authors’ contributions:

**ZYW and YWX** conceived and designed research.

**ZYW and SYH** collected data and conducted research.

**ZYW and SYH** wrote the initial paper.

**JDY and DPL** revised the paper.

**ZYW and YWX** had primary responsibility for final content.

All authors read and approved the final manuscript.

## References

[1] Ata B, Seyhan A, Seli E (2019). Diminished ovarian reserve versus ovarian aging: overlaps and differences. Curr Opin Obstet Gynecol.

[2] Scantamburlo VM, Linsingen RV, Centa LJR, Toso KFD, Scaraboto D, Araujo Junior E (2021). Association between decreased ovarian reserve and poor oocyte quality. Obstet Gynecol Sci.

[3] Yücel B, Kelekci S, Demirel E (2018). Decline in ovarian reserve may be an undiagnosed reason for unexplained infertility: A cohort study. Arch Med Sci.

[4] Rasool S, Shah D (2017). Fertility with early reduction of ovarian reserve: the last straw that breaks the Camel's back. Fertil Res Pract.

[5] Grisendi V, Mastellari E, La Marca A (2019). Ovarian Reserve Markers to Identify Poor Responders in the Context of Poseidon Classification. Front Endocrinol (Lausanne).

[6] Siddiqui QUA, Anjum S, Zahra F, Yousuf SM (2019). Ovarian reserve parameters and response to controlled ovarian stimulation in infertile patients. Pak J Med Sci.

[7] Practice Committee of the American Society for Reproductive Medicine (2020). Electronic address: asrm@asrm.org;Practice Committee of the American Society for Reproductive Medicine. Testing and interpreting measures of ovarian reserve: a committee opinion. Fertil Steril.

[8] Zhang HH, Xu PY, Wu J, Zou WW, Xu XM, Cao XY (2014). Dehydroepiandrosterone improves follicular fluid bone morphogenetic protein-15 and accumulated embryo score of infertility patients with diminished ovarian reserve undergoing in vitro fertilization: a randomized controlled trial. J Ovarian Res.

[9] Devine K, Mumford SL, Wu M, DeCherney AH, Hill MJ, Propst A (2015). Diminished ovarian reserve in the United States assisted reproductive technology population: diagnostic trends among 181,536 cycles from the Society for Assisted Reproductive Technology Clinic Outcomes Reporting System. Fertil Steril.

[10] Wang X, Jin L, Mao YD, Shi JZ, Huang R, Jiang YN (2021). Evaluation of Ovarian Reserve Tests and Age in the Prediction of Poor Ovarian Response to Controlled Ovarian Stimulation-A Real-World Data Analysis of 89,002 Patients. Front Endocrinol (Lausanne).

[11] Huang Y, Li J, Zhang F, Liu Y, Xu G, Guo J (2018). Factors affecting the live-birth rate in women with diminished ovarian reserve undergoing IVF-ET. Arch Gynecol Obstet.

[12] Goldman MB, Thornton KL, Ryley D, Alper MM, Fung JL, Hornstein MD (2014). A randomized clinical trial to determine optimal infertility treatment in older couples: The Forty and Over Treatment Trial (FORT-T). Fertil Steril.

[13] Sapet C, Gavoille A, Sesques A, Freour T, Subtil F, Salle B (2021). Results of in vitro fertilization versus intrauterine insemination in patients with low anti-Müllerian hormone levels. A single-center retrospective study of 639 +119 cycles. J Gynecol Obstet Hum Reprod.

[14] Sun H, Gong TT, Jiang YT, Zhang S, Zhao YH, Wu QJ (2019). Global, regional, and national prevalence and disability-adjusted life-years for infertility in 195 countries and territories 1990-2017: Results from a global burden of disease study 2017. Aging (Albany NY).

[15] Ozturk S, Sut HK, Kucuk L (2019). Examination of sexual functions and depressive symptoms among infertile and fertile women. Pak J Med Sci.

[16] Ozturk R, Taner A, Guneri SE, Yilmaz B (2017). Another face of violence against women: Infertility. Pak J Med Sci.

[17] Alam F, Khan TA, Amjad S, Rehman R (2019). Association of oxidative stress with female infertility - A case control study. J Pak Med Assoc.

[18] Rehman R, Rajpar HI, Ashraf M, Iqbal NT, Lalani S, Alam F (2020). Role of oxidative stress and altered thyroid hormones in unexplained infertility. J Pak Med Assoc.

[19] Carson SA, Kallen AN (2021). Diagnosis and Management of Infertility: A Review. JAMA.

[20] Allahbadia GN (2017). Intrauterine Insemination: Fundamentals Revisited. J Obstet Gynaecol India.

[21] Diamond MP, Legro RS, Coutifaris C, Alvero R, Robinson RD, Casson P (2015). Letrozole, Gonadotropin, or Clomiphene for Unexplained Infertility. N Engl J Med.

[22] Gungor ES, Seval O, Ilhan G, Verit FF (2018). Effect of intrauterine insemination treatment on sexual function and quality of life for infertile women. Pak J Med Sci.

[23] Farquhar CM, Liu E, Armstrong S, Arroll N, Lensen S, Brown J (2018). Intrauterine insemination with ovarian stimulation versus expectant management for unexplained infertility (TUI): a pragmatic, open-label, randomised, controlled, two-centre trial. Lancet.

[24] Nandi A, Bhide P, Hooper R, Gudi A, Shah A, Khan K (2017). Intrauterine insemination with gonadotropin stimulation or in vitro fertilization for the treatment of unexplained subfertility: a randomized controlled trial. Fertil Steril.

[25] Chang Y, Li J, Li X, Liu H, Liang X (2018). Egg Quality and Pregnancy Outcome in Young Infertile Women with Diminished Ovarian Reserve. Med Sci Monit.

[26] Cimadomo D, Fabozzi G, Vaiarelli A, Ubaldi N, Ubaldi FM, Rienzi L (2018). Impact of Maternal Age on Oocyte and Embryo Competence. Front Endocrinol (Lausanne).

[27] Shahine LK, Marshall L, Lamb JD, Hickok LR (2016). Higher rates of aneuploidy in blastocysts and higher risk of no embryo transfer in recurrent pregnancy loss patients with diminished ovarian reserve undergoing in vitro fertilization. Fertil Steril.

